# Biocomposite Based on Polylactic Acid and Rice Straw for Food Packaging Products

**DOI:** 10.3390/polym16081038

**Published:** 2024-04-10

**Authors:** Piyaporn Kampeerapappun, Narongchai O-Charoen, Pisit Dhamvithee, Ektinai Jansri

**Affiliations:** 1 Faculty of Textile Industries, Rajamangala University of Technology Krungthep, Bangkok 10120, Thailand; piyaporn.k@mail.rmutk.ac.th; 2 Department of Materials and Metallurgical Engineering, Faculty of Engineering, Rajamangala University of Technology Thanyaburi, Pathumthani 12110, Thailand; narongchai.o@en.rmutt.ac.th; 3 Division of Food Science and Nutrition, Faculty of Agricultural Product Innovation and Technology, Srinakharinwirot University, Nakhon Nayok 26120, Thailand; pisitd@g.swu.ac.th; 4 Division of Polymer Materials Technology, Faculty of Agricultural Product Innovation and Technology, Srinakharinwirot University, Nakhon Nayok 26120, Thailand

**Keywords:** biocomposite, food packaging, sustainability, waste straw, polylactic acid

## Abstract

Plastic containers, commonly produced from non-biodegradable petroleum-based plastics such as polyethylene (PE), polypropylene (PP), and polyethylene terephthalate (PET), raise significant environmental concerns due to their persistence. The disposal of agricultural waste, specifically rice straw (RS), through burning, further compounds these environmental issues. In response, this study explores the integration of polylactic acid (PLA), a biodegradable material, with RS using a twin-screw extruder and injection process, resulting in the creation of a biodegradable packaging material. The inclusion of RS led to a decrease in the melt flow rate, thermal stability, and tensile strength, while concurrently enhancing the hydrophilic properties of the composite polymers. Additionally, the incorporation of maleic anhydride (MA) contributed to a reduction in the water absorption rate. The optimized formulation underwent migration testing and met the standards for food packaging products. Furthermore, no MA migration was detected from the composite. This approach not only provides a practical solution for the disposal of RS, but also serves as an environmentally-friendly alternative to conventional synthetic plastic waste.

## 1. Introduction

Recently, Thailand has faced significant challenges in the management of rice straw (RS) after harvesting. Current management methods vary, and include collection using a baler for sale, plowing it into the soil, and incineration. Each method has its own advantages and disadvantages depending on labor and cost. However, the most popular method is burning, as it is the easiest, fastest, and has a relatively lower cost. The ensuing impacts include the degradation of cultivated land, and the destruction of organic matter and soil nutrients, leading to the generation of smoke, ash, and micro-dust, and, most significantly, contributing to climate change [1,2]. This issue is a very important problem that should be resolved not only in Thailand, but throughout the world. Moreover, particulate matter (PM) from biomass burning is emitted into the air, affecting air quality and human health, leading to cardiovascular morbidities, respiratory symptoms, and increased adult mortality in high-risk groups [3,4,5].

RS can be utilized diversely, for example, in animal feed, as a substrate for mushroom cultivation, as biomass, and for biochar production [6]. Additionally, it can be mixed with polymers to produce various products such as automotive components, artificial wood, and composite films [7,8,9,10,11,12]. RS can help increase the strength of the polymer when it has an appropriate particle size and is mixed in proportion [13,14,15,16]. Although the use of RS as reinforcement in composites is a good alternative way to utilize it, new problems may arise when these composites are discarded. They are scarcely recycled due to the difficulty of the recycling process. Therefore, with sustainability in mind, the production of RS-based composites should involve the use of biodegradable materials as the matrix.

Polylactic acid (PLA) is a bio-based polymer that naturally degrades under suitable conditions. It is a highly favored biodegradable polymer due to its excellent mechanical properties, low thermal expansion coefficient, and versatility in forming various products [17]. In previous studies, Mat Zubir et al. [18] used a two-roller mill mixer to prepare a composite material consisting of PLA and RS to study the effect of different ratios of RS on the mechanical properties of PLA/RS composites. It was found that the content of RS fibers increased from 5% to 25%, causing the tensile strength and elongation of the composites to decrease, while the Young’s modulus increased. Some researchers employed pretreatment methods on RS before it is mixed with PLA, utilizing various substances. For example, Liao et al. [19] ground RS and treated it with NaOH for 24 h, followed by washing with distilled water until it reached neutrality. Subsequently, it was mixed with PLA and PEG using an internal mixer at RS ratios of 10, 20, and 30 wt%, and subjected to mechanical property testing. The results indicated that the introduction of RS fibers did not lead to any improvement in the mechanical properties of the PLA matrix. However, the tensile strength of the composite increased as the RS fiber content increased from 10% to 30%. Yu et al. [20] discovered that using 120 μm sized RS particles, which underwent pretreatment with an alkali, ultrasound techniques, and a combination of both when mixed with PLA at a 1:99 mass ratio through a twin-screw extruder, can significantly increase the matrix and particles’ adhesion in all cases. Furthermore, this composite material exhibited reduced water absorption compared to PLA/RS composites without pretreatment. Additionally, PLA-g-MA was utilized as a compatibilizer for the composite, and some studies employed both pretreated RS and PLA-g-MA. Nyambo et al. [21] observed enhancements in tensile and flexural strength, with increases of approximately 20% and 14%, respectively, in PLA mixed with 30% wheat straw composites upon the addition of 3 and 5 phr PLA-g-MA compared to those without the addition of maleic anhydride. These improvements were attributed to the strong interfacial adhesion between the fibers and the matrix, which contributed to the observed increase in strength. Asheghi-Oskooee et al. [22] identified the optimal processing conditions for preparing PLA-g-MA and incorporating it into a composite of PLA with treated RS. The mechanical properties, such as Young’s modulus, tensile strength, impact strength, and tensile toughness, of PLA mixed with pretreated RS and PLA-g-MA, or without PLA-g-MA, showed an increase compared to the PLA/RS composite.

This research was designed to simplify the raw material preparation process, minimizing chemical usage, in contrast to the complex methods of pretreatment or grafting typically employed. This approach allows farmers to actively engage in material preparation. Compostable packaging products, incorporating PLA reinforced by RS, were subjected to migration testing for food contact materials. However, the addition of maleic anhydride (MA), a colorless crystalline substance, into the PLA matrix for compatibility or plasticization purposes could pose hazards to humans. Therefore, an assessment was conducted to test the release of MA from the packaging.

## 2. Materials and Methods

### 2.1. Materials

The RS variety of Pathum Thani 1 was obtained from Kamphaeng Phet, Thailand. It was collected immediately after harvest and dried in the sun for 3 days. An initial quantity of 200 g of RS was reduced in size using a Retsch SM100 cutting mill (Retsch, Haan, Germany) for 10 min, followed by sieving with a mesh size of 300 μm. After grinding, the RS was spongy, soft, lightweight, and spreads easily in the air. The matrix material used in this study was polylactic acid (PLA), grade 4043D, a product of NatureWorks LLC derived from PTT Global Chemical PCL (Bangkok, Thailand). It has a melt flow rate of 6.00 g/10 min under the conditions of 210 °C/2.16 kg, a peak melt temperature ranging from 145 °C to 160 °C, a glass transition temperature between 55 °C and 60 °C, and a specific gravity of 1.24 g/cm^3^. Maleic anhydride (MA), obtained from Tokyo Chemical Industry Co., Ltd., Tokyo, Japan, with a molecular weight of 98.06 and a purity greater than 99.0%, was employed as a compatibilizer and/or plasticizer in the composite.

### 2.2. PLA/RS Composite Preparation

PLA and RS were placed in a UF-260 Memmert universal oven (Memmert, Schwabach, Germany) at 80 °C for 6 h to remove moisture before use. PLA/RS composite pellets were prepared using an E101 twin-screw extruder (Chareon Tut Co., Ltd., Samutprakarn, Thailand) within a temperature range of 100–170 °C from the feed section to the die section, with a screw speed of 80 rpm. The mixing ratios between PLA and RS were 90:10, 80:20, and 70:30, represented as PLA/RS10, PLA/RS20, and PLA/RS30, respectively. The same process was repeated while incorporating 1% (phr) MA content, resulting in PLA/RS10MA, PLA/RS20MA, and PLA/RS30MA. Then, an injection machine was employed to produce the test specimens, with the processing temperature set at 100–190 °C from the feed section to the nozzle section.

### 2.3. Characterizations

#### 2.3.1. Melt Flow Index

An XRL-400A melt flow index tester (C.B.N. Material Test Co., Ltd., Samutprakarn, Thailand) was utilized to determine the melt flow index (MFI), which indicates the rheology of materials. The test data were presented as weight (g) per 10 min. Testing conditions adhered to the ASTM D1238 standard [23], with a temperature of 210 °C and a load of 2.16 kg. Neat PLA was processed with a twin-screw extruder under the same conditions as described in Section 2.2, to ensure comparable results with the other samples.

#### 2.3.2. Particle Size Distribution

The particle size distribution was measured with a Mastersizer 3000 laser diffraction particle size analyzer (Malvern Instruments, Malvern, UK) using the dry analysis method. A total of 3 g of RS powder was fed into the system at a feed rate of 25% and a pressure of 2 bar until a laser obscuration rate of 0.49% was reached. A particle refractive index of 1.540 and a particle absorption index of 0.01 were utilized. The sample underwent five consecutive runs, yielding the average particle size as well as the parameters Dv10, Dv50, and Dv90.

#### 2.3.3. Mechanical Properties

The tensile, flexural, and Izod impact strength were established for evaluation of the mechanical properties of the materials in this research. Tensile strength was determined according to the ASTM D638 standard [24], using a 3400 series universal testing machine (Instron (Thailand) Ltd., Bangkok, Thailand) with a cross-head speed of 50 mm/min. The outcomes were reported in terms of tensile strength (MPa) and tensile modulus (GPa). Similarly, flexural strength (three-point) was assessed following the ASTM D790 guidelines [25], employing a machine with a cross-head speed of 2 mm/min. The results were reported in terms of flexural strength (MPa). The Izod impact strength assessment was conducted according to ASTM D256 [26], utilizing an impact pendulum model 9050 (Instron (Thailand) Ltd., Bangkok, Thailand). V-notched Izod specimens were employed in the experiment, with the results expressed in units of kJ/m^2^.

#### 2.3.4. Morphology Analysis

The RS powders were examined at 5× magnification using an Eclipse LV100N POL polarized light microscope (Nikon Instruments, Tokyo, Japan). The analysis was performed using NIS-Elements v.4.0 software to evaluate particle size and shape.

After the tensile test, the specimens were examined for the distribution and homogeneity of the reinforcement in the matrix using a JSM-6510LV scanning electron microscope (SEM) (Jeol Ltd., Tokyo, Japan). The sputter coater was utilized to enhance conductivity by covering the specimens with gold on their cross-sections. An accelerating voltage of 10 kV was employed for SEM image analysis of the biocomposites.

#### 2.3.5. Thermal Behavior

A DSC 25 differential scanning calorimeter (DSC) (TA Instrument, New Castle, DE, USA) was used to analyze the thermal transitions of materials. The analysis involved a three-step operation sequence. In the first step, the specimen was heated at a rate of 10 °C/min, progressing from 0 °C to 200 °C; it was then held for 5 min to eliminate thermal history, resulting in the first heat curve. Subsequently, in the second step, the specimen was cooled down at the same rate as the heating step, from 200 to 0 °C, yielding the cooling curve. In the last step, the specimen was reheated at the same rate until it reached 200 °C, producing the second heat curve.

The values of the melting temperature transition (T_m_), glass temperature transition (T_g_), and enthalpy for calculating the percentage of crystallinity (X_c_) of the materials were determined from the second heat curve. Equation (1) was then applied to estimate the X_c_ value.

(1) $$X_{c}\left(\%\right)=100\times \frac{∆H_{m}-∆H_{c}}{∆H_{f}\times X_{PLA}}$$

where 

∆H_m_ is the melting enthalpy;

∆H_c_ is the crystallization enthalpy;

∆H_f_ is the melting enthalpy of 100% crystalline PLA (93 J/g);

X_PLA_ represents the PLA weight fraction.

The correlation between temperature and weight loss of the materials was investigated using a TGA 550 thermogravimetric analyzer (TA Instruments, DE, USA) in a nitrogen atmosphere. The heating rate was 10 °C/min, and the specimen weighed between 3 and 5 g. The temperature range covered was from 50 °C to 600 °C.

#### 2.3.6. Water Absorption Test

The water absorption test of the material was in accordance with ASTM D570 standards [27]. The specimens were prepared with a diameter of approximately 50 mm and a thickness of 3.2 mm. They were then dried at 50 °C in an oven for 24 h and weighed (W_0_). Subsequently, they were placed in a container with distilled water maintained at 23 °C for 24 h. Afterwards, the water on the surface of the specimens was quickly removed with a dry cloth, and they were immediately reweighed (W_t_). The water absorption capacity (WA) was calculated as a percentage using Equation (2).

(2) $$WA\left(\%\right)=100\times \frac{W_{t}-W_{0}}{W_{0}}$$

where 

WA is the water absorption percentage;

W_0_ is the starting weight;

W_t_ is the specimen’s weight at time t.

#### 2.3.7. Migration Testing for Food Contact Materials (FCMs)

The composite with the highest strength was chosen as the representative for testing. The test is divided into an analysis of lead and cadmium content according to methods for food analysis of the Department of Medical Sciences (DMSc) and the National Bureau of Agricultural Commodity and Food Standards (ACFS) [28]. The test results must not detect the presence of either substance. Another test was conducted to measure the residue remaining from evaporation according to JETRO 2008 standards and testing methods for implements, containers, and packaging [29]. Specific migration testing applies either to an individual substance or to a group of similar substances. The established limits are based on the toxicological hazard posed by each substance. Test conditions were determined to replicate actual operating conditions at temperatures lower than 100 °C, specifically set at 60 °C for 30 min. Four types of solvents were used as food simulants: distilled water (for foods with a pH of more than 5), 4% acetic acid (for foods with a pH of less than 5), 20% ethanol (for foods containing alcohol), and n-heptane (for food containing fat or oil).

#### 2.3.8. Gas Chromatography-Mass Spectrometry (GC-MS)

The determination of MA was conducted using Agilent 7000C Triple Quadrupole static headspace GC-MS/MS with 7890B GC (Agilent Technologies, Inc., Santa Clara, CA, USA), operating in electron ionization mode for ion generation. The HP-5ms GC column (30 m × 0.25 mm with 0.25 μm thickness) was utilized for the analysis.

To begin, 50 mg of the sample was placed in 20 mL headspace glass-sealed vials, followed by extraction at 60 °C for 30 min. Injection utilized a split mode with a ratio of 20:1, with the injection port held at 300 °C, and a sample volume of 1 mL.

The column temperature was initially set at 40 °C for 3 min, then ramped up at a rate of 4 °C/min until reaching 200 °C. Subsequently, it was further increased to 280 °C at a rate of 20 °C/min and held at this temperature for 3 min. Helium served as the carrier gas, flowing at a constant rate of 1.0 mL/min. Molecular ions within the mass range of 29 to 500 *m*/*z* (mass-to-charge ratio) were analyzed for identification purposes.

## 3. Results and Discussion

### 3.1. Melt Flow Index

**Figure 1:**
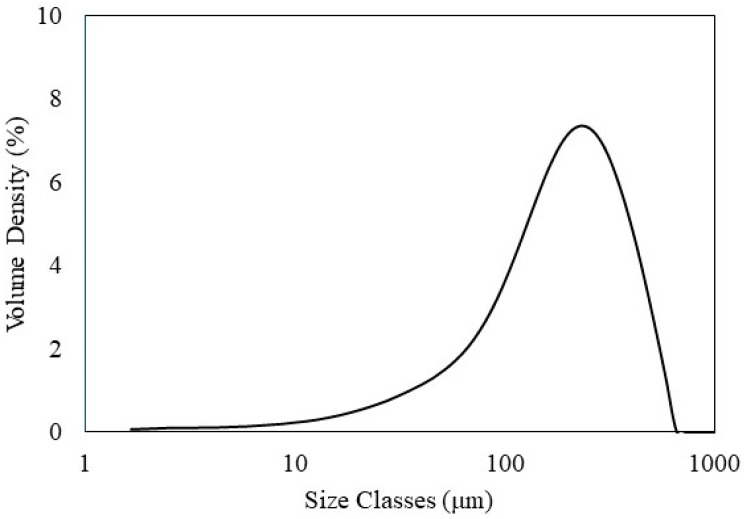
Particle size distribution of RS.

The MFI results are shown in Table 1 and Figure 1. It was discovered that the flow rate decreased as the RS content increased, suggesting that RS obstructed the flow of molten PLA [30]. However, the melt flow properties of the biocomposite were improved by adding MA, as it functions as a lubricant [31], which facilitates movement under load.

### 3.2. Particle Size Distribution

The RS was reduced in size using a cutting mill and passed through a sieve with a mesh size of 300 μm. The particle size distribution of RS before the formation of the composite is depicted in Figure 1.

**Figure 2:**
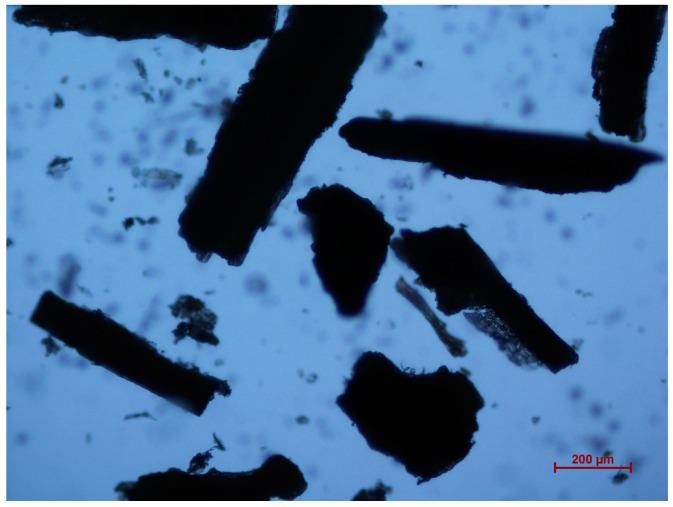
Morphology of RS powder.

The average particle size of RS was 223.87 µm, with the particle size distribution represented by Dv10 = 50.58 µm, Dv50 = 201.48 µm, and Dv90 = 431.38 µm (Figure 1). These Dv values indicate that 10%, 50%, and 90% of the RS particles are below the corresponding micron sizes, respectively. For instance, Dv90 = 431.38 µm means that 90% of the RS particles are below 431.38 µm in size. The RS particle size could be larger than the opening size of the sieve because RS particles are rod-shaped, with lengths greater than their diameters (Figure 2). Therefore, these particles can pass through the sieve [32].

### 3.3. Mechanical Properties

**Figure 3:**
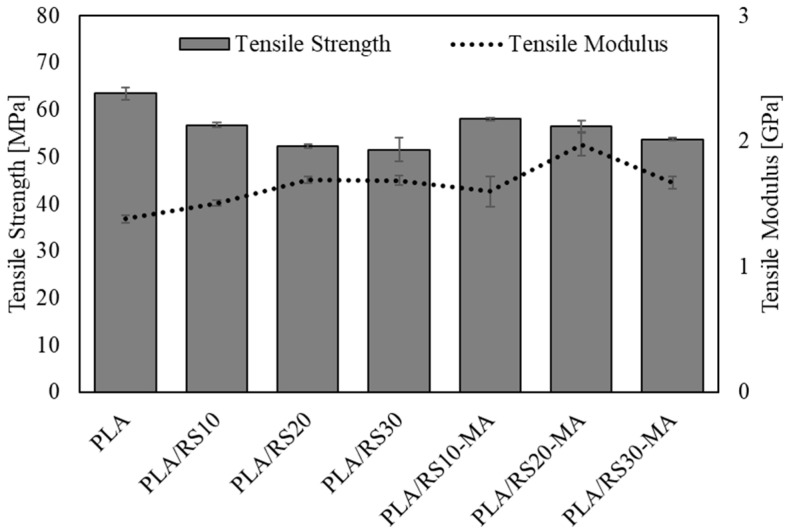
Tensile strength and tensile modulus of PLA, PLA/RS composites, and PLA/RS-MA composites.

The results of tensile properties are shown in Figure 3 and Table 2. It was found that neat PLA exhibited the highest tensile strength at 63.47 MPa and the lowest tensile modulus at 1.38 GPa. However, in the cases of the biocomposites, an increase in the RS content led to a decrease in tensile strength in both composites with MA and without MA. This decrease can be attributed to the incompatibility between the matrix and reinforcement. Specifically, PLA/RS10-MA exhibited the highest tensile strength at 58.08 MPa, while PLA/RS30 showed the lowest tensile strength at 51.57 MPa. On the other hand, the tensile modulus increased with increasing RS content, up to 20%, in the cases of the composites both with MA and without MA. This increase can be attributed to the RS particles acting as rigid fillers, which do not melt and thus increase the rigidity of PLA during processing [18].

At the same time, RS particles also insert themselves between PLA molecular chains, inhibiting movement and causing the composite to lose its flexibility [20]. The composite PLA/RS20-MA had the highest modulus of 1.97 GPa. In the case of 30% RS content, the tensile modulus decreased in both cases because the RS particles were unevenly distributed. The results of the comparison of the composites with MA and without MA clearly show that MA acts as a plasticizer or compatibilizer that helps RS disperse well in PLA [31], resulting in higher tensile properties than composites without MA in all cases.

**Figure 4:**
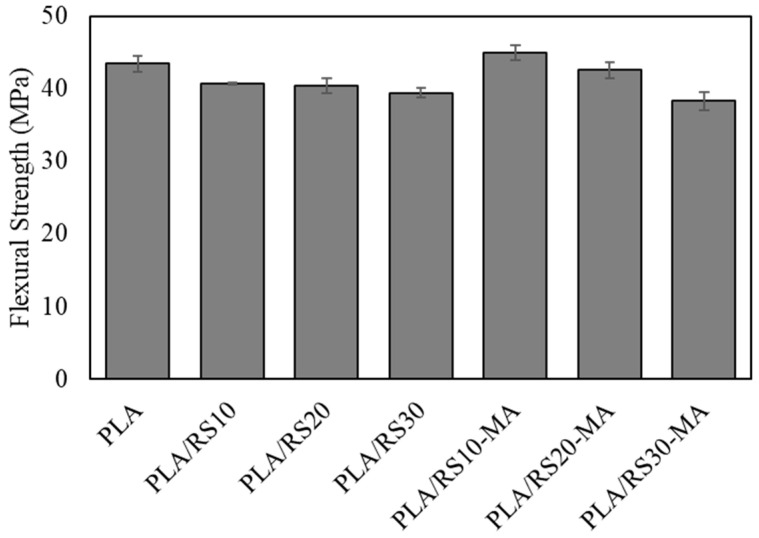
Flexural strength of PLA, PLA/RS composites, and PLA/RS-MA composites.

The results of the flexural strength showed a similar trend to the tensile strength, as shown in Figure 4 and Table 2. It was found that neat PLA had a flexural strength of 43.45 MPa, which is higher than that of the other composites except for PLA/RS10-MA. The flexural strength of the composites decreased with increasing RS content in all cases. The comparison of the composite with MA and without MA showed that adding MA helped increase flexural strength, except in the case of PLA/RS30-MA. This exception is likely a result of the inconsistent ratio of MA to RS, leading to excessive RS content, clustering of RS particles, increased surface roughness of the specimen, and an uneven distribution of force received from the loading head.

**Figure 5:**
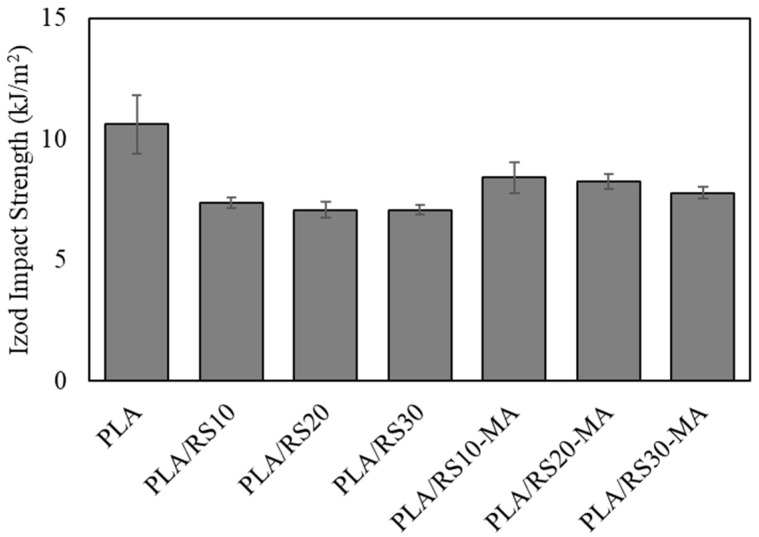
Izod impact strength of PLA, PLA/RS composites, and PLA/RS-MA composites.

The Izod impact strength is related to the force exerted on the embedded particles to dislodge them from the matrix, and is an important energy dissipation mechanism in particle-reinforced composites. It was found that neat PLA had the highest impact strength at 10.6 kJ/m^2^, while the PLA/RS10-MA composite had the highest at 8.40 kJ/m^2^ (Figure 5 and Table 2). The Izod impact strength of the composites decreased with increasing RS content in cases both with and without MA. Furthermore, the addition of MA resulted in a higher Izod impact strength than without MA in all cases. These results align with the findings of the tensile strength test regarding the rigidity of the filler and the incompatibility, including the dispersion of RS particles.

### 3.4. Morphology Inspection

**Figure 6:**
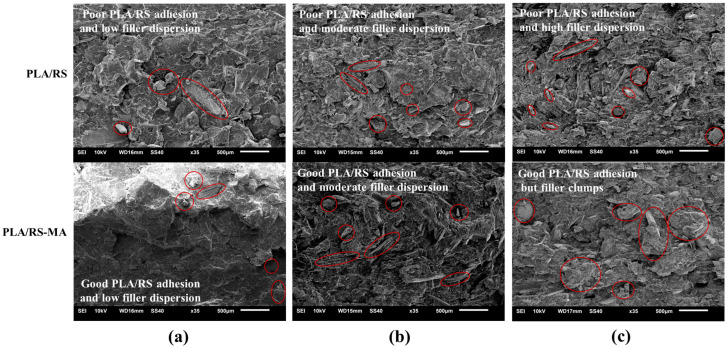
SEM micrographs of PLA/RS composites: (**a**) PLA/RS10, (**b**) PLA/RS20, and (**c**) PLA/RS30.

The distribution of RS particles on the cross-section of the specimens was investigated with SEM, as shown in Figure 4. As expected, the composites exhibited a clear indication of poor interphase between the PLA matrix and RS particles, suggesting incompatibility between RS particles and PLA. There was a noticeable increase in phase separation as the RS content increased, as depicted in Figure 6a–c. The results of the addition of MA showed that it could help the RS particles merge well with PLA. This was evident from the RS particles embedded in the PLA matrix, demonstrating a strong interphase between the phases. Because MA is a coupling agent that reduces the hydrophilicity of the material, it increases the adhesion force between phases. Moreover, it enhanced the dispersion of the filler, thereby increasing the dimension stability of the composites [33]. However, it is essential that the addition of MA corresponds to the content of filler or reinforcement [34]. In the case of PLA/RS30-MA, there appeared to be an inconsistency between the MA and RS content, resulting in uneven distribution and some clumping, thereby leading to mechanical properties that do not meet the desired standard.

### 3.5. Thermal Properties

**Figure 7:**
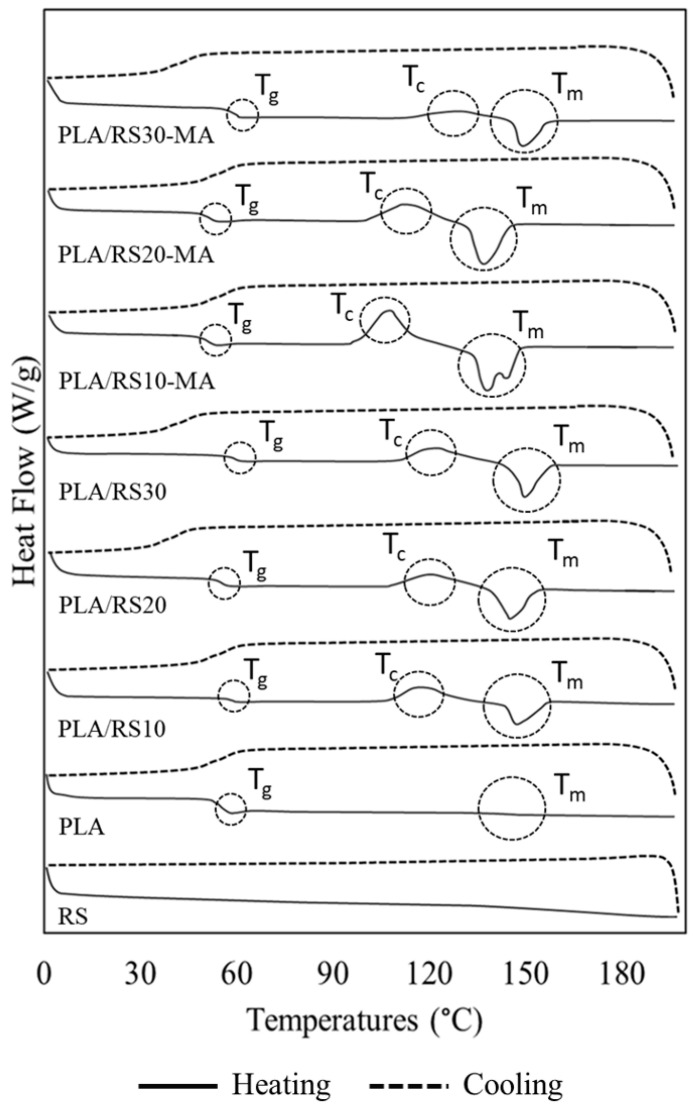
DSC thermograms of PLA, PLA/RS composites, and PLA/RS-MA composites.

DSC measurements of neat PLA and PLA/RS composites are presented in Figure 7, with transition temperatures and calculated X_c_ values provided in Table 3 for clarity.

In the case of the composites without MA, T_g_ values were similar to those of neat PLA in the temperature range of 61–62 °C. Increasing the RS content caused the T_g_ to increase until the RS content reached 20%, after which it decreased slightly. Meanwhile, in the case of the composites with MA, T_g_ was lower than in both neat PLA and composites without MA by approximately 3–5 °C. This was attributed to the better distribution and loose agglomeration of RS particles. Consequently, the chains could move more easily, facilitating crystallization in all cases [35]. In terms of the effect of the RS ratio on the T_c_ value, it was observed that in the case of the composites without MA, there was a non-trend fluctuation in the temperature range of 120–128 °C. However, in the case of the composites with MA, the T_c_ showed a noticeable increase with increasing RS content, measuring 114.16, 122.99, and 130.22 °C, respectively. It is worth noting that in the case of neat PLA, T_c_ was absent, which may be attributed to the use of a high cooling rate, possibly interfering with the crystallization mechanisms. Considering the effect of the RS content on the T_m_, it was observed that in the case of the composites without MA, there was no trend fluctuation in the range of 146–149 °C. However, in the case of the composites with MA, there was a noticeable increase in T_m_ with increasing RS content, measuring 145.99, 147.61, and 152.01 °C, respectively.

The calculation of percent crystallinity (%X_c_) revealed that neat PLA had the lowest value at 0.12, while PLA/RS30-MA had the highest at 2.97. These results clearly demonstrated that %X_c_ increased with an increase in the RS content, suggesting that RS particles could easily induce crystallization in the composite. When comparing composites with and without MA, the values were 0.43, 1.41, and 1.44 for the former and 2.06, 2.79, and 2.97 for the latter, respectively. This indicates that MA enhances the composite’s %X_c_, possibly by improving compatibility and aiding in better dispersion of the RS particles [36]. Furthermore, Figure 7 indicates that almost all materials exhibited a single melting peak, except in the case of PLA/RA10-MA, which displayed a double melting peak. This phenomenon may be attributed to the presence of two distinct crystal structures undergoing melting [37,38,39].

**Figure 8:**
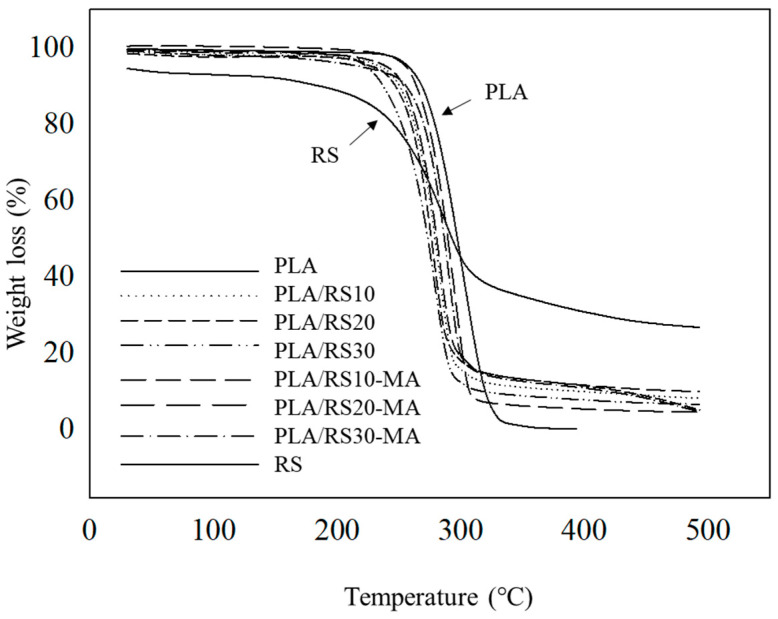
TGA thermograms of PLA, RS, PLA/RS composites, and PLA/RS-MA composites.

The relationship between increasing temperature and the weight loss of the composites was assessed using TGA, as depicted in Figure 8. In all cases, PLA, RS, and PLA/RS composites exhibited one-step degradation. Table 3 illustrates that PLA demonstrated the highest thermal stability within a decomposition temperature range of 293.07–342.32 °C, with a weight loss of 99.63%. Conversely, RS exhibited decomposition within a temperature range of 264.04–337.42 °C, with the lowest weight loss value recorded at 69.16%, likely attributable to the ash fraction content after decay. In the case of the composite materials, it was found that the decomposition temperature decreased with increasing RS content within the temperature range of 272.65–326.25 °C. This phenomenon is expected, as the higher thermal stability of the PLA content decreased and was replaced by RS particles with lower thermal stability [38]. Additionally, composites with MA exhibited higher thermal stability compared to those without MA, attributed to the enhanced surface adhesion between the matrix and reinforcement [39,40].

### 3.6. Water Absorption

**Figure 9:**
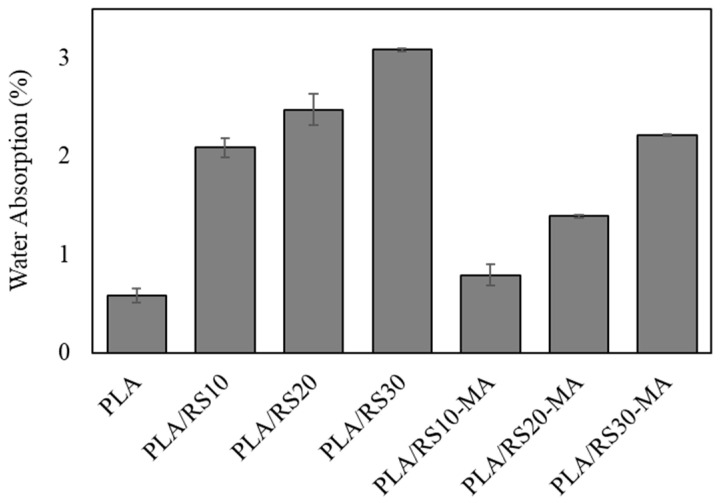
Water absorption of PLA, PLA/RS composites, and PLA/RS-MA composites.

The results of the water absorption test are presented in Figure 9. As anticipated, PLA exhibited the lowest absorption rate at 0.58%, while the composites showed an increase with rising RS content, with PLA/RS30 registering the highest value at 3.08%. This trend is attributed to RS’s main component, cellulose, which is hydrophilic [34]. When comparing cases with and without MA, it was observed that the addition of MA led to a reduction in water absorption. This can be attributed to the enhanced interfacial adhesion between RS and PLA, which limits the diffusion of water molecules into the composite [41,42]. The decrease in water absorption with the addition of MA may also be attributed to the cross-linking reaction of components. Several factors contribute to the reduction in water absorption with the addition of MA: alteration of pores within the specimens or at the interface between the matrix and reinforcement, improved bonding between the matrix interface and reinforcement [42,43], and decreased hydrophilicity due to the reduced number of OH groups, thereby reducing the opportunities for bonding with water [44,45].

### 3.7. Migration Testing for Food Contact Materials (FCMs)

**Figure 10:**
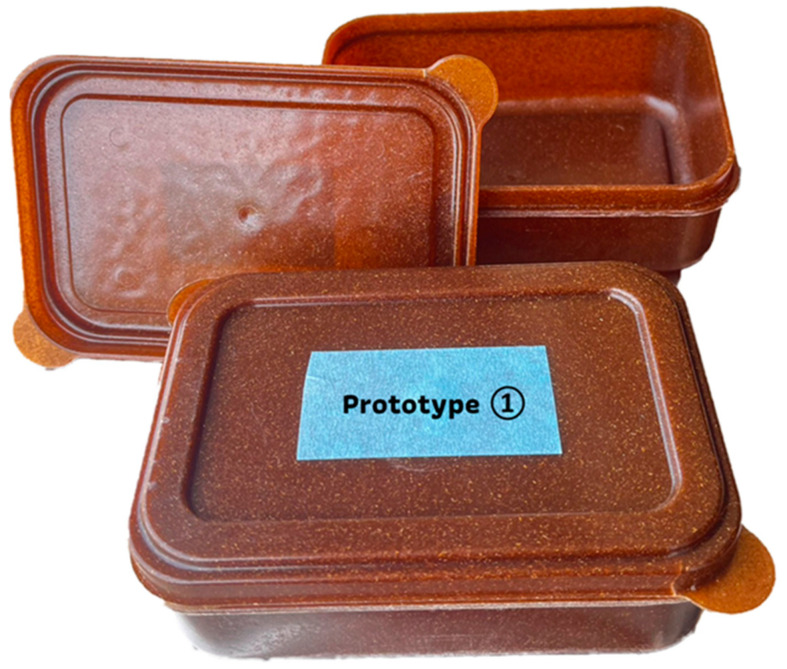
Prototype of biodegradable food containers.

The food container composite with the highest strength, PLA/RS20-MA (Figure 10), was selected for migration testing and GC-MS analysis.

The results of the plastic residue test found that there was no harmful heavy metal contamination which is prohibited in food packaging, such as cadmium and lead, in the samples (Table 4). In addition, the solubility of the chemicals from the plastic was tested at 60 °C for 30 min, followed by an analysis of the residue remaining from the evaporation process used to extract the sample. It was found that there were dissolved residues of 11.5, 7.5, and 6.8 mg/dm^3^ from the extractions with water, 4% acetic solution, and 20% ethanol solution, respectively, while no residue was found when dissolved in n-heptane at 25 °C for 60 min. Color release from the plastic was not found in any of the solutions. Overall, all test results were subjected to Thai Industrial Standards (TIS 655 Part 1-2010) [46] and the announcement by the Ministry of Public Health (No. 435, 2022) of Thailand, as shown in Table 4.

**Figure 11:**
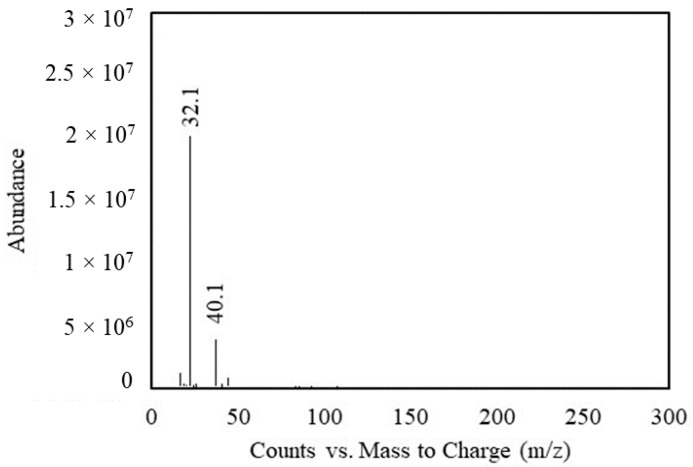
MS spectra of PLA/RS20-MA composite.

The composite underwent testing for MA release at 60 °C for 30 min using GC-MS, with the results shown in Figure 11.

The two main peaks, detected at 32.1 and 40.1 *m*/*z*, corresponded to the molecular structures of O_2_ [47] and atmospheric contaminants [48], respectively. The absence of MA detection can be attributed to the lack of specific ionization, as evidenced by the absence of the base peak at 54 and the M+ (molar mass) peak at 98 *m*/*z* [49,50].

## 4. Conclusions

In this research, the production of biocomposites from PLA mixed with RS was investigated, considering different mixing ratios ranging from 10% to 30% (wt) of RS, along with the effect of adding 1% (phr) of MA. For the preparation of the composite pellets and testing specimens, a twin-screw extruder and an injection molding machine were utilized, respectively. The study revealed that as the RS content increased, the melt flow rate, thermal stability, and tensile strength decreased, while the tensile modulus, percentage of crystallinity, and water absorption rate increased. Furthermore, the addition of MA resulted in an improvement in the melt flow rate, thermal stability, and mechanical properties, along with a decrease in the rate of water absorption. The PLA/RS20-MA composite with the highest tensile modulus was selected for migration testing for food contact materials and was found to meet the standard, compared to commonly used packaging materials such as PP, PE, and PET. Additionally, no traces of MA were detected in the composite material through GCMS analysis. This study successfully offers a new alternative for mitigating environmental pollution caused by the burning of rice straw, contributing to the fight against global warming. It highlights the potential for producing biodegradable composites from PLA and rice straw for use as environmentally-friendly food containers. Future developments will focus on studying the biodegradability of PLA/RS biocomposites.

## Figures and Tables

**Table 1:** Melt flow index of PLA, PLA/RS composites, and PLA/RS-MA composites.

Samples	Melt Flow Index (g/10 min) (210 °C/2.16 kg)
PLA (Processed)	25.37 (±1.14)
PLA/RS10	21.74 (±0.64)
PLA/RS20	18.80 (±0.60)
PLA/RS30	15.90 (±1.17)
PLA/RS10-MA	23.51 (±0.45)
PLA/RS20-MA	19.01 (±0.39)
PLA/RS30-MA	17.77 (±1.27)

**Table 2:** Mechanical properties of PLA, PLA/RS composites, and PLA/RS-MA composites.

Samples	Tensile Strength (MPa)	Tensile Modulus (GPa)	Flexural Strength (MPa)	Izod Impact Strength (kJ/m^2^)
PLA	63.47 (±1.27)	1.38 (±0.03)	43.45 (±1.08)	10.6 (±1.21)
PLA/RS10	56.76 (±0.46)	1.51 (±0.02)	40.67 (±0.09)	7.36 (±0.23)
PLA/RS20	52.30 (±0.43)	1.69 (±0.03)	40.40 (±0.96)	7.06 (±0.32)
PLA/RS30	51.57 (±2.60)	1.69 (±0.04)	39.40 (±0.65)	7.06 (±0.18)
PLA/RS10-MA	58.08 (±0.31)	1.60 (±0.12)	44.93 (±1.04)	8.40 (±0.63)
PLA/RS20-MA	56.53 (±1.25)	1.97 (±0.09)	42.83 (±1.08)	8.23 (±0.30)
PLA/RS30-MA	53.73 (±0.31)	1.67 (±0.05)	38.27 (±1.18)	7.76 (±0.23)

**Table 3:** Thermal properties of PLA and PLA/RS composites.

Samples	Differential Scanning Calorimetry (DSC)				Thermogravimetric Analysis (TGA)		
	T_g_ (°C)	T_c_ (°C)	T_m_ (°C)	Crystallinity (%X_c_)	Weight Loss (%)	Temperature Decomposition (T_onset_) (°C)	Temperature Decomposition (T_peak_) (°C)
PLA	61.71	-	148.05	0.12	99.63	293.07	342.32
RS	-	-	-	-	69.16	264.04	337.42
PLA/RS10	62.44	120.04	149.94	0.43	97.76	281.19	314.93
PLA/RS20	62.64	128.45	146.35	1.41	95.98	278.34	313.20
PLA/RS30	61.60	121.96	148.87	1.44	93.19	272.65	312.81
PLA/RS10-MA	58.33	114.16	145.99	2.06	95.13	291.08	326.25
PLA/RS20-MA	57.58	122.99	147.61	2.79	94.29	293.42	324.00
PLA/RS30-MA	61.77	130.22	152.01	2.97	86.85	281.77	314.55

**Table 4:** Migration test.

Food Simulant Substitutes	Residue Content (mg/dm^3^)	Color Release (mg/dm^3^)	Maximum Permitted Concentrations (mg/dm^3^) *
Distilled water	11.5	-	<30
4% acetic acid	7.5	-	<30
20% ethanol	6.8	-	<30
n-heptane (25 °C/60 min)	-	-	-

* According to the maximum permitted concentrations of polypropylene, polyethylene, and polyethylene terephthalate.

## Data Availability

Data are contained within the article.

## References

[1] Dobermann A., Fairhurst T. (2002). Rice straw management. Better Crops Int..

[2] Ibrahim R., Sapuan S., Ilyas R., Atikah M. (2021). Utilization of rice straw as a raw material for food packaging. Bio-Based Packaging: Material, Environmental and Economic Aspects.

[3] Suriyawong P., Chuetor S., Samae H., Piriyakarnsakul S., Amin M., Furuuchi M., Hata M., Inerb M., Phairuang W. (2023). Airborne particulate matter from biomass burning in Thailand: Recent issues, challenges, and options. Heliyon.

[4] Buya S., Lim A., Saelim R., Musikasuwan S., Choosong T., Taneepanichskul N. (2024). Impact of air pollution on cardiorespiratory morbidities in Southern Thailand. Clin. Epidemiol. Glob. Health.

[5] Xing Y.F., Xu Y.H., Shi M.H., Lian Y.X. (2016). The impact of PM2.5 on the human respiratory system. J. Thorac. Dis..

[6] Goodman B.A. (2020). Utilization of waste straw and husks from rice production: A review. J. Bioresour. Bioprod..

[7] Bassyouni M., Hasan S.W.U. (2015). The use of rice straw and husk fibers as reinforcements in composites. Biofiber Reinforcements in Composite Materials.

[8] Kitisatorn W. (2023). Mechanical and Sound Absorption of Rice Straw Fiber Reinforced Bio-Epoxy Composites for Lightweight Components in Rail Vehicles. Mater. Sci. Forum.

[9] Saidah A., Susilowati S.E., Nofendri Y. (2019). Effect of fiber loading and alkali treatment on rice straw fiber reinforced composite for automotive bumper beam application. Int. J. Adv. Sci. Eng. Inf. Technol..

[10] Tawfik M.E., Eskander S., Nawwar G.A. (2017). Hard wood-composites made of rice straw and recycled polystyrene foam wastes. J. Appl. Polym. Sci..

[11] Xu H., Dun M., Zhang Z., Zhang L., Shan W., Wang W. (2022). A New Process of Preparing Rice Straw-Reinforced LLDPE Composite. Polymers.

[12] Zhang L., Xu H., Wang W. (2020). Performance of Straw/Linear Low Density Polyethylene Composite Prepared with Film-Roll Hot Pressing. Polymers.

[13] Zhang X., Wang Z., Cong L., Nie S., Li J. (2020). Effects of fiber content and size on the mechanical properties of wheat straw/recycled polyethylene composites. J. Polym. Environ..

[14] Zhang W., Chen J., Bekele L.D., Liu Y., Duns G.J., Jin L. (2016). Physical and mechanical properties of modified wheat straw-filled polyethylene composites. BioResources.

[15] Kuang X., Kuang R., Zheng X., Wang Z. (2010). Mechanical properties and size stability of wheat straw and recycled LDPE composites coupled by waterborne coupling agents. Carbohydr. Polym..

[16] Pan M.Z., Zhou D.G., Bousmina M., Zhang S. (2009). Effects of wheat straw fiber content and characteristics, and coupling agent concentration on the mechanical properties of wheat straw fiber-polypropylene composites. J. Appl. Polym. Sci..

[17] Farah S., Anderson D.G., Langer R. (2016). Physical and mechanical properties of PLA, and their functions in widespread applications—A comprehensive review. Adv. Drug Deliv. Rev..

[18] Mat Zubir N.H., Ting S.S., Santiagoo R., Noimam N., Wang J. (2016). Tensile properties of rice straw fiber reinforced poly (lactic acid) biocomposites. Adv. Mater. Res..

[19] Liao Z.F., Song G.L., Shi F., Yin Z.S., Yang Y., Niu Z., Tang G.Y. (2011). Preparation and characterization of pla/rice straw fiber composite. Appl. Mech. Mater..

[20] Yu W., Dong L., Lei W., Zhou Y., Pu Y., Zhang X. (2021). Effects of rice straw powder (RSP) size and pretreatment on properties of FDM 3D-printed RSP/poly (lactic acid) biocomposites. Molecules.

[21] Nyambo C., Mohanty A.K., Misra M. (2011). Effect of maleated compatibilizer on performance of PLA/wheat Straw-Based green composites. Macromol. Mater. Eng..

[22] Asheghi-Oskooee R., Morsali P., Mohammadi-Roshandeh J., Hemmati F. (2024). Tailoring interfacial adhesion and mechanical performance of biocomposites based on poly(lactic acid)/rice straw by using maleic anhydride through reactive extrusion process. J. Appl. Polym. Sci..

[23] (2016). For Melt Flow Rates of Thermoplastics by Extrusion Plastomer.

[24] (2016). Standard Test Method for Tensile Properties of Plastics.

[25] (2016). Standard Test Methods for Flexural Properties of Unreinforced and Reinforced Plastics and Electrical Insulating Materials.

[26] (2016). Standard Test Methods for Determining the Izod Pendulum Impact Resistance of Plastics.

[27] (2016). Standard Test Method for Water Absorption of Plastics.

[28] Kānphǣt T.K.W., National Bureau of Agricultural Commodity and Food Standards (2003). Compendium of Methods for Food Analysis /cDepartment of Medical Sciences and National Bureau of Agriculture Commodity and Food Standards.

[29] Japan External Trade Organization (2008). Specifications, Standards and Testing Methods for Foodstuffs, Implements, Containers and Packaging, Toys, Detergents.

[30] Charoen N., Kampeerapappun P., Charoenlarp K., Petchwattana N., Jansri E. (2022). Green composites based on PLA and cotton fabric waste: Preparation and characterization. Recycling.

[31] Clasen S.H., Müller C.M., Pires A.T. (2015). Maleic anhydride as a compatibilizer and plasticizer in TPS/PLA blends. J. Braz. Chem. Soc..

[32] Freitas P.A.V., González-Martínez C., Chiralt A. (2020). Application of ultrasound pre-treatment for enhancing extraction of bioactive compounds from rice straw. Foods.

[33] Kim H.-S., Lee B.-H., Choi S.-W., Kim S., Kim H.-J. (2007). The effect of types of maleic anhydride-grafted polypropylene (MAPP) on the interfacial adhesion properties of bio-flour-filled polypropylene composites. Compos. Part A Appl. Sci. Manuf..

[34] Lv S., Gu J., Tan H., Zhang Y. (2016). Modification of wood flour/PLA composites by reactive extrusion with maleic anhydride. J. Appl. Polym. Sci..

[35] Dominguez-Candela I., Gomez-Caturla J., Cardona S., Lora-Garcia J., Fombuena V. (2022). Novel compatibilizers and plasticizers developed from epoxidized and maleinized chia oil in composites based on PLA and chia seed flour. Eur. Polym. J..

[36] Jang H., Kwon S., Kim S.J., Park S.-i. (2022). Maleic anhydride-grafted PLA preparation and characteristics of compatibilized PLA/PBSeT blend films. Int. J. Mol. Sci..

[37] Petchwattana N., Channuan W., Naknaen P., Narupai B. (2019). 3D printing filaments prepared from modified poly (lactic acid)/teak wood flour composites: An investigation on the particle size effects and silane coupling agent compatibilisation. J. Phys. Sci..

[38] Petchwattana N., Covavisaruch S., Chanakul S. (2012). Mechanical properties, thermal degradation and natural weathering of high density polyethylene/rice hull composites compatibilized with maleic anhydride grafted polyethylene. J. Polym. Res..

[39] Azmin S.N.H.M., Nor M.S.M. (2020). Development and characterization of food packaging bioplastic film from cocoa pod husk cellulose incorporated with sugarcane bagasse fibre. J. Bioresour. Bioprod..

[40] Bascón-Villegas I., Pereira M., Espinosa E., Sánchez-Gutiérrez M., Rodríguez A., Pérez-Rodríguez F. (2022). A new eco-friendly packaging system incorporating lignocellulose nanofibres from agri-food residues applied to fresh-cut lettuce. J. Clean. Prod..

[41] Varghese S.A., Pulikkalparambil H., Promhuad K., Srisa A., Laorenza Y., Jarupan L., Nampitch T., Chonhenchob V., Harnkarnsujarit N. (2023). Renovation of Agro-Waste for sustainable food packaging: A Review. Polymers.

[42] Chang S.Y., Ismail H., Ahsana Q. (2012). Effect of maleic anhydride on kenaf dust filled polycaprolactone/thermoplastic sago starch composites. Bioresources.

[43] Manaia J.P., Manaia A. (2021). Interface modification, water absorption behaviour and mechanical properties of injection moulded short hemp fiber-reinforced thermoplastic composites. Polymers.

[44] Manaia J.P., Manaia A.T., Rodriges L. (2019). Industrial hemp fibers: An overview. Fibers.

[45] Mutjé P., Vallejos M., Girones J., Vilaseca F., López A., López J., Méndez J. (2006). Effect of maleated polypropylene as coupling agent for polypropylene composites reinforced with hemp strands. J. Appl. Polym. Sci..

[46] (2010). Thai Industrial Standard, Plastic Utensils for Food—Part 1 Polyethylene, Polypropylene, Polystyrene, poly(ethylene terephthalate), poly(vinyl alcohol) and poly(methyl pentene); TIS 655 (Part 1-2010). https://www.jetro.go.jp/thailand/e_survey/_493154.html.

[47] National Center for Biotechnology Information (2024). PubChem Compound Summary for CID 977, Oxygen. https://pubchem.ncbi.nlm.nih.gov/compound/Oxygen.

[48] Gao Y., Walker M.J., Barrett J.A., Hosseinaei O., Harper D.P., Ford P.C., Williams B.J., Foston M.B. (2018). Analysis of gas chromatography/mass spectrometry data for catalytic lignin depolymerization using positive matrix factorization. Green Chem..

[49] National Center for Biotechnology Information (2024). PubChem Compound Summary for CID 7923, Maleic Anhydride. https://pubchem.ncbi.nlm.nih.gov/compound/Maleic-Anhydride.

[50] NIST Mass Spectrometry Data Center (2014). Maleic Anhydride Mass Spectrum. https://webbook.nist.gov/cgi/cbook.cgi?ID=C108316&Mask=200#Top.

